# Development of a novel anti-hepatitis B virus agent via Sp1

**DOI:** 10.1038/s41598-019-56842-9

**Published:** 2020-01-08

**Authors:** Michiyo Hayakawa, Hideaki Umeyama, Mitsuo Iwadate, Y.-H. Taguchi, Yoshihiko Yano, Takashi Honda, Saori Itami-Matsumoto, Ritsuzo Kozuka, Masaru Enomoto, Akihiro Tamori, Norifumi Kawada, Yoshiki Murakami

**Affiliations:** 1 Department of Hepatology, Graduate School of Medicine, Osaka City University, Osaka, 545-8585 Japan; 2 Department of Biological Sciences, Chuo University, Tokyo, 112-8551 Japan; 3 Department of Physics, Chuo University, Tokyo, 112-8551 Japan; 4 Division of Gastroenterology, Department of Internal of Medicine, Kobe University Graduate School of Medicine, Kobe, 650-0017 Japan; 5 Division of Gastroenterology, Department of Internal Medicine, Nagoya University Graduate School of Medicine, Nagoya, 466-8550 Japan; 6 Present Address: Department of Molecular Pathology, Tokyo Medical University, 6-1-1, Shinjuku, Shinjuku-Ku, Tokyo 160-8402 Japan

**Keywords:** Hepatitis B virus, Hepatitis B

## Abstract

Nucleos(t)ide analog (NA) therapy has proven effective in treating chronic hepatitis B. However, NAs frequently result in viral relapse after the cessation of therapy. This is because NAs cannot fully eliminate the viral episomal covalently closed circular DNA (cccDNA) in the nucleus. In this study, we identified small molecular compounds that control host factors related to viral replication using *in silico* screening with simulated annealing based on bioinformatics for protein-ligand flexible docking. Twelve chemical compound candidates for alpha-glucosidase (AG) inhibitors were identified from a library of chemical compounds and used to treat fresh human hepatocytes infected with HBV. They were then monitored for their anti-viral effects. HBV replication was inhibited by one candidate (1-[3-(4-tert-butylcyclohexyl)oxy-2-hydroxypropyl]-2,2,6,6-tetramethylpiperidin-4-ol) in a dose-dependent manner. This compound significantly reduced ccc DNA production, compared to Entecavir (*p* < 0.05), and had a lower anti-AG effect. Gene expression analysis and structural analysis of this compound showed that its inhibitive effect on HBV was via interaction with Sp1. The nuclear transcription factor Sp1 acts on multiple regions of HBV to suppress HBV replication. Identifying candidates that control nuclear transcription factors facilitate the development of novel therapies. Drugs with a mechanism different from NA are promising for the elimination of HBV.

## Introduction

Nucleos(t)ide analogs (NAs) are a standard first-line therapy for chronic hepatitis B (CHB). NAs cause only mild side effects while effectively inhibiting viral replication; however, the frequency of relapse after patients cease therapy is high. Novel agents with different mechanisms that can overcome these deficits are needed to improve the efficacy of CHB therapy.

Alpha-glucosidase inhibitors (AGI) are used to reduce post-prandial glucose levels in patients with type 2 diabetes mellitus^1^. Many animal viruses are constructed of an outer envelope composed of one or more viral glycoproteins. Viral glycoproteins identify and bind to receptor sites on the host’s membrane and affect other critical functions necessary to maintain the viral life cycle, such as virion assembly, secretion, and viral infectivity. In particular, AGI regulates the replication of human immunodeficiency virus (HIV)^2^, HBV^2–5^, and influenza^6,7^ by targeting the N-glycan pathway.

HBV has a complicated life cycle. It infects hepatocytes via sodium taurocholate co-transporting polypeptide (NTCP)^8^ following the uncoating of the viral shell; then, relaxed circular (RC) DNA is transported into the nucleus. The RCDNA is converted to cccDNA; then, four HBV-related RNAs are transcribed from cccDNA^9^. These RNAs are exported to the cytoplasm, then pregenomic RNA, one of the four RNAs, is reverse transcribed into (−) stranded DNA. Finally, RCDNA is synthesized from (−) stranded DNA, packed into a shell, then released externally as virus particles^10^.

The treatment goals for CHB are to decrease the morbidity and mortality related to HBV infection. The long-term suppression of HBV replication has improved inflammation and fibrosis symptoms in histological findings. Given the persistence of cccDNA in the nucleus of hepatocytes, even in persons with serological markers of resolved infection, there is a lifelong risk of reactivation of the infection. A virological cure is defined as the eradication of a virus, including its cccDNA^11,12^. However, this is not currently an attainable goal^13^. Importantly, NAs predominantly target the cytoplasmic reverse transcription of the pregenomic RNA but do not target the episomal persistence of cccDNA in the cell nucleus. In order to develop a new virus elimination method, it is necessary to analyze the pathway of synthesis of cccDNA and host factors related to virus elimination. This study also focused on Sp1, one of the nuclear transcription factors involved in HBV replication. Sp1 can bind to the core promoter region of HBV^14^ and suppress the function of the HBV HBx protein (HBx)^15^.

This study searched for novel antiviral candidates that suppressed host-virus replication factors using *in silico* screening.

## Results

### The procedure of *in silico* screening for alpha-glucosidase inhibitors

*In silico* screening was used to select 3089 chemical compounds from the 2,200,000 contained in the AKos database. From this group, 12 alpha-glucosidase inhibitor compounds were selected based on their binding strength and structural similarity. The antiviral activity of these 12 candidates was determined, in addition to three alpha-glucosidase inhibitors (Acarbose (AGI1), Miglitol (AGI2), and Voglibose (AGI3)) used to treat type 2 diabetes (Table 1). AGI6 and AGI13 have different functional groups; however, their structures have identical skeletons. AGI13 (Ghose-Crippen-Viswanadhan octanol-water partition coefficient (ALOGP) = 3.25) is more hydrophobic than AGI6 (ALOGP = 2.85). Since the two compounds have similar structures and physicochemical properties, the strength of human AG activity is considered the result of the fitting compatibility of the two compounds on the AG receptor (Supplementary Fig. 1).

**Table 1:** List of the chemical compound.

Code.No	Name of substrate	MW/compositional formula	
AGI1	Acarbose	660.1/C25H43NO18	
AGI2	Miglitol	207.2/C8H17NO5	
AGI3	Voglibose	267.3/C10H21NO7	
	Name of substrate/AKOS No/Ranking No.	MW/compositional formula/FPAS score	**Structural formula**
AGI4	2,6-dimethyl-1-[2-(oxolan-2-ylmethoxy)ethyl]piperidine/AKOS016935375/231	242.3/C15H30NO/531.5	
AGI5	1-(4-methylpiperidin-1-yl)-3-naphthalen-2-yloxypropan-2-ol/AKOS016182856/236	300.4/C16H34N2O/610.3	
AGI6	1-(2,6-dimethylpiperidin-1-yl)-3-(3-methoxyphenoxy)propan-2-ol/AKOS016050483/154	326.5/ C14H28NO2/586.1	
AGI7	1-(4-tert-butylcyclohexyl)oxy-3-(2,6-dimethylpiperidin-1-yl)propan-2-ol/AKOS016286797/142	326.5/ C17H28NO3/586.1	
AGI8	1-(1-adamantylmethoxy)-3-(3,5-dimethylpiperidin-1-yl)propan-2-ol/AKOS016318436/172	336.5/C16H34N2O/554.0	
AGI9	2-(1,3-dioxolan-2-yl)-1-(2-methoxyethyl)piperidine/AKOS007774093/123	216.3/C14H25N2O/528.8	
AGI10	3-[2-(1,3-dioxolan-2-yl)piperidin-1-yl]propan-1-ol/AKOS007869050/131	216.3/C15H30NO3/481.3	
AGI11	1-methoxy-3-[4-(4-methylcyclohexyl)piperazin-1-yl]propan-2-ol/AKOS016924865/33	272.4/C15H24NO3/511.7	
AGI12	[2-[[1-(2,2-difluoroethyl)piperidin-4-yl] amino]cycloheptyl]methanol/AKOS016984232/8	292.4/C14H30N2O/539.4	
AGI13	1-[3-(2-chlorophenoxy)-2-hydroxypropyl]-2,2,6,6-tetramethylpiperidin-4-ol/AKOS016128493/13	342.9/C15H30F2N2O/531.6	
AGI14	1-[3-(4-tert-butylcyclohexyl)oxy-2-hydroxypropyl]-2,2,6,6-tetramethylpiperidin-4-ol/AKOS016287378/5	370.6/C14H28NO4/543.4	
AGI15	1-[2-hydroxy-3-(3,3,5-trimethylcyclohexyl)oxypropyl]-2,2,6,6-tetramethylpiperidin-4-ol/AKOS016287379/6	356.6/ C15H32N2O2/542.1	

### Screening for anti-HBV activity and cell toxicity of human alpha-glucosidase inhibitor candidates

**Figure 1:**
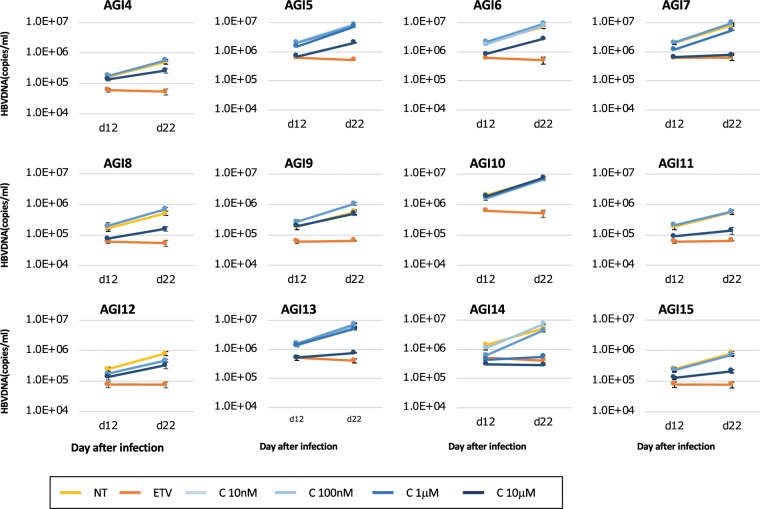
The anti-viral effect of alpha-glucosidase inhibitor candidates. The vertical and horizontal axis shows the amount of HBV-DNA (copies/mL) and days after infection, respectively. The standard deviation is also shown. Each experiment was repeated three times.

**Figure 2:**
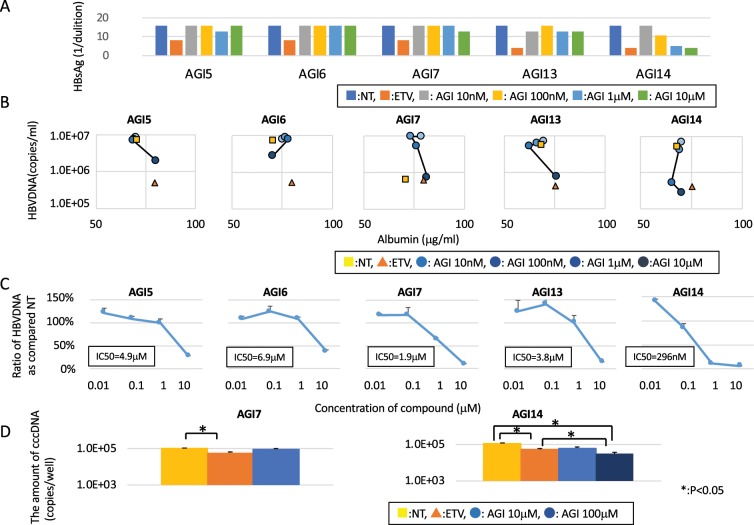
Alpha-glucosidase inhibitor candidates’ effect on HBV replication. (**A**) HBsAg level after treatment with alpha-glucosidase inhibitor candidates. HBsAg levels in the supernatant for corresponding alpha-glucosidase inhibitor candidates on day 22. The vertical axis depicts the dilution level of HBsAg in the medium. (**B**) The relationship between HBV-DNA and human albumin. The line chart and bar graph show the amount of HBV-DNA (right vertical axis) and the relative amount of HBsAg (left vertical axis), respectively. (**C**) The relationship between HBV-DNA and the concentration of alpha-glucosidase inhibitor candidates. The vertical and horizontal axis is the ratio of HBV-DNA in non-treated samples and the concentration of alpha-glucosidase inhibitor candidates, respectively. Each IC_50_ is also described. (**D**) The level of cccDNA in PXB cells. An asterisk indicates a significant difference (*p* < 0.05). Each experiment was repeated three times.

PXB cells infected with HBV were used to observe anti-viral activity and cell toxicity. AGI1-AGI3 and AGI4-AGI15 were dissolved in H_2_O and DMSO, respectively. AGI10 was not used in this study because it was insoluble in DMSO and H_2_O. Entecavir (ETV) was used as a positive control for the anti-HBV drug. AGI1-AGI3 showed no observable anti-viral effect or cell toxicity (Supplementary Fig. 2). AGI5, AGI6, AGI7, AGI13, and AGI14 reduced the concentration of HBV-DNA in the supernatant in a dose-dependent manner (Fig. 1 and Supplementary Table 1). AGI14 reduced HBsAg in the supernatant in a dose-dependent manner; however, there was no noticeable reduction following treatment with AGI5, AGI6, AGI7, and AGI13 (Fig. 2A). There was also no noticeable change in human albumin concentration in the supernatant following treatment with these five AGI candidates (Fig. 2B). The IC_50_ of AGI5, AGI6, AGI7, AGI13, and AGI14 were 4.9 μM, 6.9 μM, 1.9 μM, 3.8 μM, and 296 nM, respectively (Fig. 2C). Based on the value of their IC_50_, AGI7 and AGI14 were selected for analysis of anti-cccDNA activity. The amount of cccDNA remaining after treatment with high concentrations of AGI14 without cell toxicity was significantly lower than that treated with ETV (Fig. 2D). Multiple experiments were conducted at different times, as described in the figure legends, confirming the reproducibility of the experimental results.

### AG activity in novel alpha-glucosidase inhibitor candidates

The ability of five novel candidates to inhibit alpha-glucosidase was verified. It was observed that AGI5, AGI6, and AGI13 mildly suppressed the activity of alpha-glucosidase in a dose-dependent manner, and conversely, the suppressive effect of AGI7 and AGI14 increased in a dose-dependent manner (Supplementary Fig. 3).

### Investigating the mechanism of the alpha-glucosidase inhibitor candidates on HBV replication

**Figure 3:**
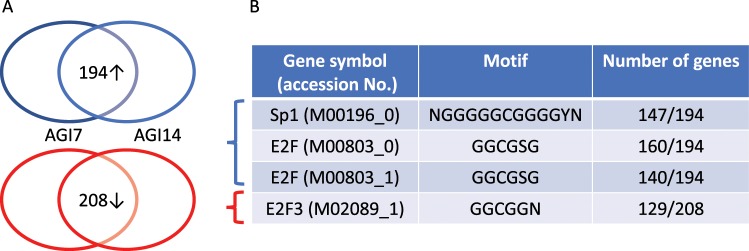
Gene expression analysis with treatment of alpha-glucosidase inhibitor candidates. (**A**) Venn diagram for detecting commonly differentially expressed genes. The upper figure shows the expression of 194 genes that were commonly upregulated in PXB cells after treatment with AGI7 or AGI14, compared to non-treated cells. The lower figure shows the expression of 208 genes that were commonly downregulated in PXB cells after treatment with AGI7 or AGI14, compared with non-treated cells. (**B**) The G-profiler analysis showed that 147, 160, and 140 genes from commonly upregulated genes recognized the promoter region of NGGGGGCGGGGYN (M00196_0), GGCGSG (M00803_0), and GGCGSG (M00803_1), respectively, and 129 genes from commonly upregulated genes also recognized the promoter region of GGCGGN (M02089_1).

Since the novel candidates had little inhibitory effect on alpha-glucosidase, an alternative mechanism of the anti-viral activity of these compounds was investigated. Gene expression patterns among non-treated cells, AGI7-treated cells, and AGI14-treated cells were compared using a next-generation sequencer (NGS). Principal component analysis (PCA) showed that 194 and 208 genes in PXB cells were commonly increased and decreased when treated with AGI7 and AGI14, respectively (Fig. 3A)^16^. To clarify the function of these genes, a G-profiler was used to analyze which regulatory DNA elements were controlled by gene clusters with common expression patterns (https://biit.cs.ut.ee/gprofiler/index.cgi).

The G-profiler showed that the GGCGSG motifs of the two E2F genes (M00803_0) and (M00803_1) were regulated by 160 and 140 of the 194 genes that were upregulated, respectively. The GGCGGGN motif of the E2F3 genes (M02089_1) was also regulated by 129 of the 208 genes that were downregulated. The NGGGGGCGGGGYN motif of Sp1 (M00196_0) was also regulated by 147 of the commonly downregulated genes (Supplementary Table 2). Since many genes recognize the promoter region of E2F3 and Sp1, it was concluded that AGI7 and AGI14 controlled viral replication via E2F3 or Sp1 (Fig. 3B).

### The interaction of AGI7 and/or AGI14 with Sp1

**Figure 4:**
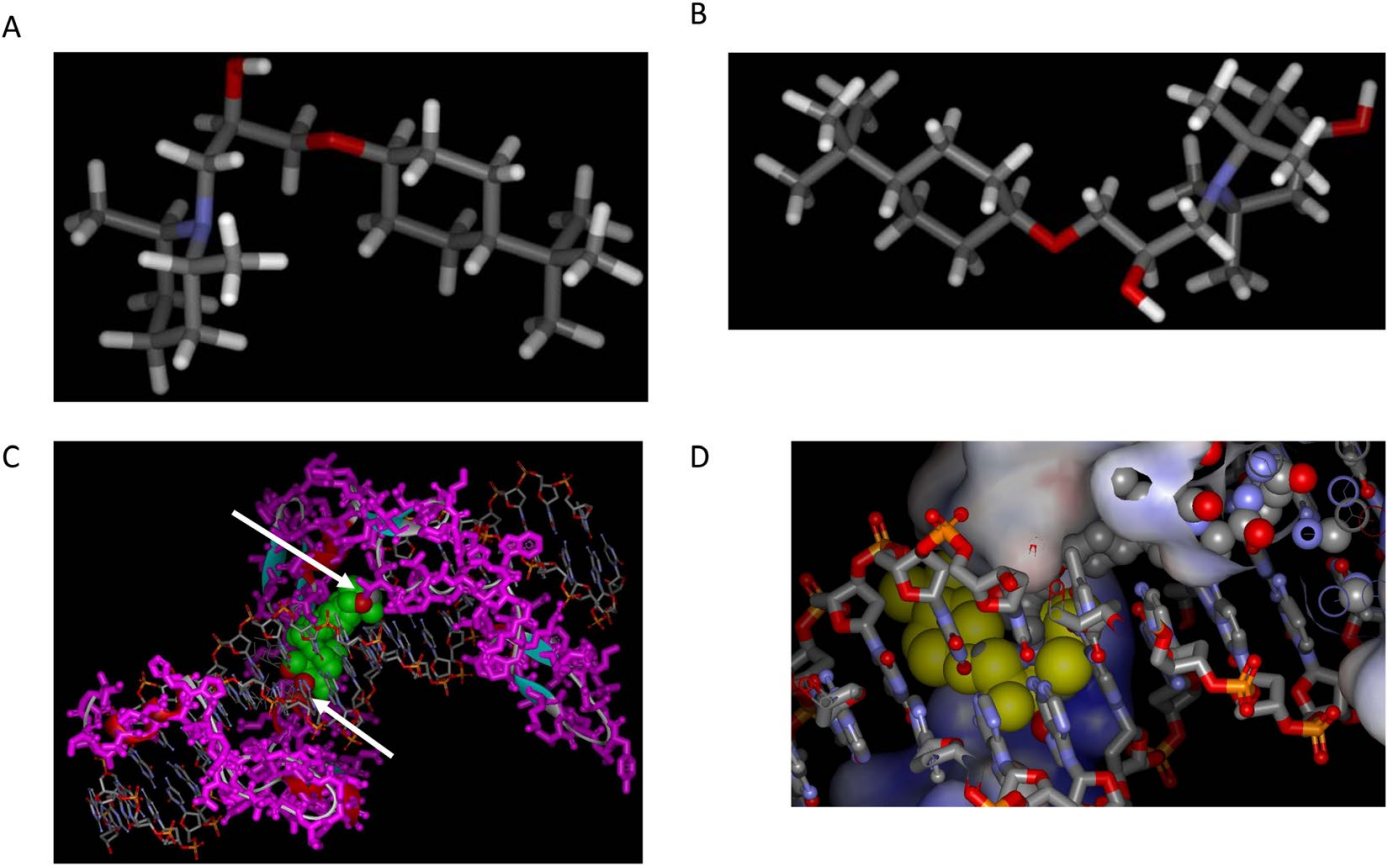
The binding status of AG7, AGI14, and Sp1. (**A**) A stick model of AGI7. (**B**) A stick model of AGI14. (**C**) The binding status of AGI14 and Sp1. Four energetically stable molecules of AGI14 (green CPK model) were docked in the interface between the DNA (stick model) and Sp1 protein (colored magenta) using two glycerol fingerprints (white arrowhead). (**D**) The interconnection of AGI7 and AGI14 to Sp1. AGI7 is depicted in the large yellow CPK model, AGI14 is depicted in the small CPK model. This whole connected model indicates that the AGI7 and AGI14 binding sites to Sp1 were different.

Sp1 has a binding motif for E2F1 (amino acids 102–125) contained within amino acids 622–668. Both E2F2 and E2F3 in the N-terminal domains, which have sequences similar to E2F1, interacted with Sp1^17,18^. Based on the gene expression and G-profiler analysis, AGI7 and AGI14 were hypothesized to have a binding affinity for Sp1. Therefore, a structural analysis was performed to confirm how AGI7 and AGI14 interact with Sp1. Using the LD method for AGI14 construction, docking between AGI14 and Sp1 was interfaced with two glycerol fingerprints^19^. However, while AGI7 could also be docked to the Sp1 protein via two molecules of glycerol, the binding site was different from that of AGI14 (Fig. 4).

### The anti-viral effect of siRNA for Sp1 and E2F3

We investigated whether siRNA that targets Sp1 and E2F3 controlled HBV replication in Hep38.7 cells. Due to a higher gene-introduction efficiency, a gene knockdown experiment was performed with Hep38.7 cells instead of PXB cells. It was observed that AGI7 and AGI14 did not inhibit HBV replication in Hep38.7 cells (data not shown). Administering siRNA for Sp1 significantly inhibited HBV replication; however, siRNA for E2F3 did not show inhibition of viral replication (Supplementary Fig. 4).

## Discussion

In this study, novel anti-viral agents that potentially control replication intermediates via nuclear transcription factors were identified. Alpha-glucosidase inhibitors are standard drugs for treating diabetes; they also exhibit the potential to act against HBV^2–5^. The novel alpha-glucosidase inhibitor candidates exhibited little inhibition of alpha-glucosidase. Assuming that they had different mechanisms for inhibiting viral replication, how a novel candidate modified gene expression was clarified. Interestingly, most genes that had a similar expression pattern after treatment with AGI7 and AGI14 had a binding affinity with the Sp1 and E2F transcription factor families (E2F1, E2F2, and E2F3). SP1 and E2F families bind to each other when acting as transcription factors^17^. Structural analysis revealed that AGI7 and AGI14 had a high affinity for Sp1, and HBV replication was suppressed, even in experiments using siRNA.

In Hep38.7 cells, AGI7 and AGI14 did not exhibit anti-viral effects (data not shown) due to the difference between PXB and Hep38.7 cells. Since PXB cells do not require passage, the effect of candidate drugs using PXB cells was determined after 22 days, and therefore, they are suitable for relatively long-term studies; however, Hep38.7 cells require passage once every three days, and therefore, they are only suitable for short-term observations of anti-viral effects. The lack of passaging of PXB cells indicated that gene transfer efficiency using liposomes was very poor; therefore, experiments with siRNA in PXB cells failed to reproduce the experimental results of Hep38.7 cells.

The expression of HBV genes was regulated by several transcription factors. Sp1-binding sites in the HBV core promoter are important in regulating the transcription of the core and precore RNA^20^. An HBV-transgenic mouse study showed that cyclin D2 was upregulated in HBV-expressing cells and liver tissues. Cyclin D2 regulated HBV replication by enhancing the activity of HBV core and Sp1 promoters by targeting the transcription factor CREB2^14^. HBx upregulates C4b-binding protein α (C4BPα) by activating transcription factor Sp1, and this protects liver cancer cells from a complement attack^21^. HBx 43–154 upregulates the activity of HBV enhancer II; moreover, CCAAT/Enhancer Binding Protein (C/EBP) and Sp1 sites on enhancer II are required for enhancer II activation by HBx 43–154^15^. Association between HBx mutated HBV, and several chromatin-modifying enzymes also influenced nuclear cccDNA^22^. HBx is essential for the initiation and maintenance of transcription from cccDNA. Therefore, HBx expression levels exactly reflect HBV RNA transcription^23^.

**Figure 5:**
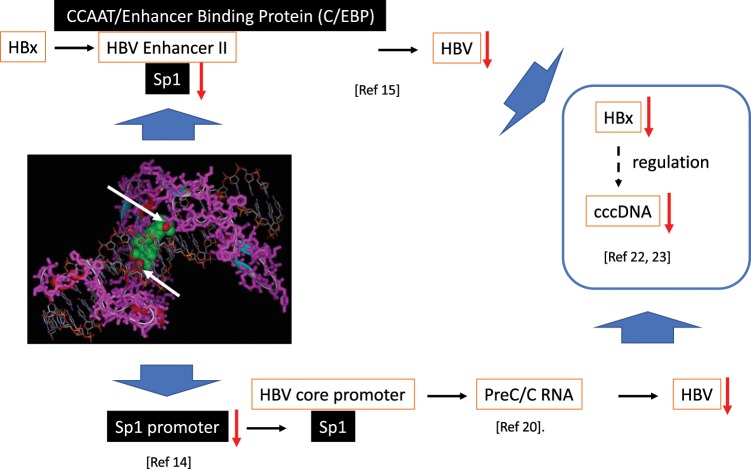
Summary of the anti-HBV effect of Sp1. AGI14 binds to Sp1 (see figure legend 4C), and as a result, Sp1 activity decreases, and HBV enhancer II initially increases in the presence of Sp1 and enhances HBV replication. However, it is assumed that HBV replication decreases due to decreased Sp1 activity. Moreover, when the promoter activity of Sp1 decreases, the activity of the HBV core promoter decreases, and it is expected that HBV replication will also decrease. Although the direct effect on cccDNA is unknown at this time, the possibility of reduced production of cccDNA with reduced viral replication capacity is also indicated.

Taken together, AGI14 bound to Sp1 and suppressed its function. Sp1 is involved in HBV replication at multiple points^15,20^. Downregulated Sp1 suppressed HBV replication via the downregulation of the HBV core promoter; the absence or silence of Sp1 suppressed HBx activity then induced the low stability of HBV episomal nuclear localization, and finally, downregulated cccDNA (Fig. 5).

## Conclusion

This study uncovered a novel class of anti-HBV agents with high anti-viral effects and low cell toxicities. These compounds did not directly target cccDNA; instead, they regulated cccDNA via nuclear transcription factors. Controlling cccDNA, which is the cause of latent infection, requires promising drugs with a low recurrence rate after the patient has completed treatment.

## Methods

### *In silico* screening and chemical compounds

*In silico* screening to develop a novel human α-glucosidase inhibitor has previously been reported^24,25^, except that here Q14697.3 in UniprotKB/Swissprot was used to analyze the amino acid sequence in the alpha-glucosidase inhibitor. A chain of PDB (protein data bank) ID 3LU4 was selected as the reference protein based on the alignment results. In total, 13 molecules were used as the fingerprints for *in silico* screening with chooseLD. The models constructed by the full automatic modeling system (FAMS) Ligand & Complex program included the following nine ligands as the fingerprint molecules: DSK (PDB ID 3L4U, A chain), ACR (2QMJ, A), KTL (3L4V, A), MIG (3L4W, A), NR3 (3L4X, A), NR4 (3L4Y, A), SSD (3L4Z, A), 3CU (3CTT, A), and BJ1 (3L4T, A). An additional four compounds (ChEMBL307429, ChEMBL111326, ChMBLE108656, and ChEMBL421040) were used. The alpha-glucosidase inhibitor activity of these compounds is registered with the ChEMBL database. Several low weight molecules were selected from the AKos database in order to fit alpha-glucosidase, the details of which have been described in previous papers^26,27^. Entecavir was purchased from Toronto Research Chemicals (Toronto, ON); AG1-AG3 and AG4-AG15 compounds were purchased from Tokyo Chemical industry (Tokyo, Japan) and AKos (Steinen, Germany), respectively (Table 1).

### *In vitro* HBV infection

The HBV-infected serum sample (genotype C) was obtained from a 54-year-old male patient. He was negative for HIV and HCV. The patient provided written informed consent, and Osaka City University Graduate School and the Faculty of Medicine’s Ethics Committee approved the study in accordance with the Helsinki Declaration 2013.

The transfection procedure, DNA extraction, quantification of HBV-DNA, HBsAg, and albumin have all been described previously^26,27^. The HBV-DNA in the sample medium was quantified by real-time qPCR (Roche Diagnostic, Tokyo, Japan) by comparing serially diluted HBV/C1.24 (HBV-DNA containing plasmid) obtained from Prof. Yasuhito Tanaka of Nagoya City University^28^.

### The quantification of cccDNA

Real-time PCR was performed with Step one plus (Applied Biosystems) using 10 μl of the sample and 20 μl of PCR mixture (Roche MasterMix, Roche Diagnostics, Almere, The Netherlands). The following target probes were used: 5′-6FAM-CGTCGCATGGARACCACCGTGAACGCC- BHQ1-3′ and IC-DNA probe: 5′-TBRCCCTTTACATCTTTCTGAAGTAGGG-3′^29^. Primer concentrations were 0.9 μM for the target and IC-DNA primers. Probe concentrations were 0.4 μM for cccDNA and 0.2 μM for IC. Amplification was performed as follows: 50 °C for 2 min, then 95 °C for 10 min, followed by 55 cycles at 95 °C for 10 sec, 58 °C for 5 sec, 63 °C for 10 sec, and 72 °C for 20 sec. Every run included a negative plasma sample, water, and a positive control with a known concentration of cccDNA (10^3^ copies/PCR)^30^.

### Alpha D-glucosidase activity assay

Where presented, IC_50_ values were determined by a non-linear, least squares regression analysis using MathIQ^TM^ (Business Solutions Ltd., UK). Where inhibition constants (Ki) are presented, the Ki values were calculated using the equation of Cheng and Prusoff^31^ using the observed IC_50_ of the tested compound, the concentration of radioligand employed in the assay, and the historical values for the K_D_ of the ligand (obtained experimentally at Eurofins Panlabs, Inc.). Where presented, the Hill coefficient (n_H_), defining the slope of the competitive binding curve, was calculated using MathIQ^TM^. Hill coefficient values significantly greater than 1.0 may suggest that the binding displacement did not follow the laws of mass action with a single binding site. Where IC_50_, Ki, and/or n_H_ data are presented without a standard error of the mean (SEM), the data were insufficient to be quantitative, and the values presented (Ki, IC_50_, n_H_) should be interpreted with caution^32^.

### Next-generating sequencing

HBV infection and alpha-glucosidase inhibitor treatment were performed in PXB cells at the same time. The medium was changed on days one and two, and PXB cells were harvested on day seven. Total RNA was extracted using a miRNeasy Mini kit (Qiagen, Hilden, Germany). Extracted RNA from each sample was quantified with a Qubit (Thermo Fisher Scientific), and was checked quality with an Agilent Bioanalyzer (Agilent Technologies, Santa Clara, CA). RNA integrity number (RIN) as RNA quality was calculated with Agilent 2100 Expert Software (Illumina, San Diego, CA). mRNA isolation, cDNA synthesis, and NGS library preparation were performed using the Illumina TruSeq Library Preparation Kit version 2 (Illumina) according to the manufacturer’s instruction. This study used between 500 and 1000 ng of total RNA for cDNA synthesis and cDNA library construction. NGS was performed on an Illumina HiSeq. 2500 instrument (Illumina).

### siRNA and cell transfection

Hep38.7 cells were kindly provided by Dr. Christophe Seeger (Fox Chase institute) and Drs. Takaji Wakita and Koichi Watashi (National Institute for Infectious Disease Research, Japan). The siRNA for Sp1 and E2F3 were synthesized in three constructs (Gene Design, Osaka, Japan). Briefly, Hep38.7 cells (2.0E + 04 cells/well) were spread into 96-well dishes, and 4 pmol of siRNA (Supplementary Table 3) and negative control siRNA (Ambion) were transfected with lipofectamine RNAiMAX (Invitrogen). Cells were harvested at 72 h after siRNA transfection.

### Statistical analysis

Two-way repeated-measures ANOVA and Dunnett’s test (SPSS, IBM, Chicago, IL) were used for statistical analysis, and statistical significance was determined as *p* < 0.05.

### Analysis for binding status among AG7, AGI14, and Sp1

Each AGI7 and AGI14 molecule was docked into the transcription factor Sp1 (UniProtKB/Swiss-Prot: P08047.3; transcription factor Sp1 of *Homo sapiens*), and two fingerprint molecules of glycerol in the PDB-code (2I13,A with 21.2% homology against the transcription factor Sp1 of *Homo sapiens*) of the PDB database were used in the docking of AGI7 and AGI14 molecules using the chooseLD program. Energetically stable conformations were noticed in the selection process of the docking structure. The protein model of transcription factor Sp1 of *Homo sapiens* was homologically modeled from 2I13,A using the FAMS Ligand & Complex program.

## Supplementary information

Supplementary information.

## Data Availability

The datasets supporting the conclusions of this article are included within the article and the additional supplementary file.
