# Author Correction: Development of a novel anti-hepatitis B virus agent via Sp1

**DOI:** 10.1038/s41598-020-63866-z

**Published:** 2020-04-21

**Authors:** Michiyo Hayakawa, Hideaki Umeyama, Mitsuo Iwadate, Y.-H. Taguchi, Yoshihiko Yano, Takashi Honda, Saori Itami-Matsumoto, Ritsuzo Kozuka, Masaru Enomoto, Akihiro Tamori, Norifumi Kawada, Yoshiki Murakami

**Affiliations:** 1 Department of Hepatology, Graduate School of Medicine, Osaka City University, Osaka, 545-8585 Japan; 2 Department of Biological Sciences, Chuo University, Tokyo, 112-8551 Japan; 3 Department of Physics, Chuo University, Tokyo, 112-8551 Japan; 4 Division of Gastroenterology, Department of Internal of Medicine, Kobe University Graduate School of Medicine, Kobe, 650-0017 Japan; 5 Division of Gastroenterology, Department of Internal Medicine, Nagoya University Graduate School of Medicine, Nagoya, 466-8550 Japan; 6 Present Address: Department of Molecular Pathology, Tokyo Medical University, 6-1-1, Shinjuku, Shinjuku-Ku, Tokyo 160-8402 Japan

Correction to: *Scientific Reports* 10.1038/s41598-019-56842-9, published online 08 January 2020

Figure 3 incorrectly shows the upregulation of the SP1 promoter motif by AGI7 and AGI14. Additionally, the accompanying legend incorrectly states that 129 and 147 genes with the E2F3 and SP1 promoter motifs, respectively, were selected from groups of commonly upregulated genes. The correct Figure 3 and its accompanying legend appears below as Figure 1.

**Figure 1:**
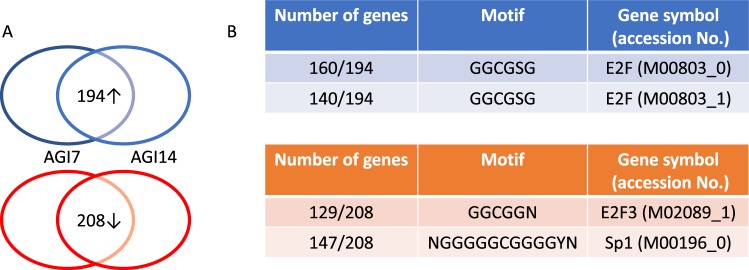
Gene expression analysis with treatment of alpha-glucosidase inhibitor candidates. (**A**) Venn diagram for detecting commonly differentially expressed genes. The upper figure shows the expression of 194 genes that were commonly upregulated in PXB cells treated with AGI7 or AGI14, compared to non-treated cells. The lower figure shows the expression of 208 genes that were commonly downregulated in PXB cells treated with AGI7 or AGI14, compared with nontreated cells. (**B**) The G-profiler analysis showed that 160, and 140 genes from commonly upregulated genes recognized the promoter region of GGCGSG (M00803_0), and GGCGSG (M00803_1), respectively, and 129 and 147 genes from commonly downregulated genes also recognized the promoter region of GGCGGN (M02089_1) and NGGGGGCGGGGYN (M00196_0), respectively.

