# Supplementary material for: Development of a novel anti-hepatitis B virus agent via Sp1
Source: Sci Rep. 2020 Jan 8;10:47. doi: 10.1038/s41598-019-56842-9 (PMC6949255; doi:10.1038/s41598-019-56842-9)
Supplement: Supplementary file 1 — Supplementary information. [file 41598_2019_56842_MOESM1_ESM.pdf]

Development of a novel anti-hepatitis B virus agent via Sp1

Michiyo Hayakawa[1], Hideaki Umeyama[2], Mitsuo Iwadate[2], Y-h Taguchi[3], Yoshihiko Yano[4], Takashi Honda[5], Saori Itami-Matsumoto[1], Ritsuzo Kozuka[1], Masaru Enomoto[1], Akihiro Tamori[1], Norifumi Kawada[1], Yoshiki Murakami[1 #*]

1. Department of Hepatology, Graduate School of Medicine, Osaka City University, Osaka 545-8585, Japan.
2. Department of Biological Sciences, Chuo University, Tokyo 112-8551, Japan.
3. Department of Physics, Chuo University, Tokyo 112-8551, Japan.
4. Division of Gastroenterology, Department of Internal of Medicine, Kobe University Graduate School of Medicine, Kobe 650-0017, Japan.
5. Division of Gastroenterology, Department of Internal Medicine, Nagoya University Graduate School of Medicine, Nagoya 466-8550, Japan.

*Corresponding author
Yoshiki Murakami M.D., Ph.D

#Present affiliation
Department of Molecular Pathology
Tokyo Medical University
6-1-1, Shinjuku, Shinjuku-Ku, Tokyo 160-8402, Japan
Tel +81-3-3351-6141
Fax +81-3-3352-6335
e-mail yoshikim@tokyo-med.ac.jp

Supplementary information

Supplementary table

Supplementary table 1. Anti-HBV effect for ETV and each alpha-glycosidase inhibitor

Supplementary table 2. List of common genes with expression level that changed with AGI7 and AGI14 treatment and the genes that recognize the promoter region of E2F, E2F2, and Sp1

Supplementary table 3. List of siRNA sequence

Supplementary figure legend

Supplementary figure 1. Estimated joint structure between alpha-glucosidase inhibitor and alpha-glucosidase

A. Estimated docking structure with Acarbose (AGI1), Miglitol (AGI2), and Voglibose (AGI3), and human alpha-glucosidase.

B. Estimated docking structure with AGI5, AGI6, AGI7, AGI13, AGI14 and human alpha-glucosidase

Supplementary figure 2. Anti-viral effect for alpha-glucosidase inhibitor for diabetes.

A. Anti-viral effect for AGI1-AGI3. Vertical and horizontal axis is the amount of HBVDNA (copies/ml) and number of days after infection.

B. Estimating cell toxicity the day after infection.

Supplementary figure 3. Suppressive effect of novel alpha-glucosidase inhibitor candidate on alpha-glycosidase

Vertical axis is inhibitory effect of alpha-glucosidase (%)

Supplementary figure 4. Anti-viral effect of siRNA for Sp1 and E2F3

Vertical axis is the relative amount of HBVDNA (normalized on negative control). TF is denoted only transfection reagent. Asterisk indicates significant difference ($p<0.05$). Each experiment was repeated three times.

Supplementary Table 1. Anti-HBV effect by ETV and each AGI (p-value between arbitrary two groups)

	d12	d22	d12	d22	d12	d22
	AGI4		AGI8		AGI13	
NT vs. ETV	1.48E-02	1.32E-03	1.48E-02	1.32E-03	4.10E-03	1.13E-03
NT vs. 10μM	6.66E-01	4.13E-01	4.76E-01	1.03E-01	3.37E-03	1.74E-03
NT vs. 100μM	3.45E-01	2.00E-02	2.43E-02	4.03E-03	3.12E-03	1.08E-03
ETV vs.10μM	5.72E-04	1.70E-04	2.03E-02	6.01E-04	8.44E-01	3.55E-03
ETV vs.100μM	2.76E-03	3.58E-03	8.44E-02	7.04E-03	9.15E-01	6.28E-01
	AGI5		AGI9		AGI14	
NT vs. ETV	1.48E-02	1.32E-03	8.72E-03	1.64E-03	4.10E-03	1.13E-03
NT vs. 10μM	1.66E-02	1.48E-03	1.07E-01	9.01E-03	4.10E-03	1.13E-03
NT vs. 100μM	2.06E-02	1.67E-03	7.62E-01	4.85E-01	3.52E-03	8.96E-04
ETV vs.10μM	3.92E-01	1.29E-01	2.88E-03	2.59E-04	4.41E-01	6.66E-01
ETV vs.100μM	3.82E-01	4.52E-01	2.31E-04	6.61E-04	5.03E-01	3.12E-02
	AGI6		AGI11		AGI15	
NT vs. ETV	1.48E-02	1.32E-03	8.72E-03	1.64E-03	4.10E-03	1.13E-03
NT vs. 10μM	1.40E-01	3.92E-02	4.50E-01	7.40E-01	6.20E-01	4.03E-01
NT vs. 100μM	2.50E-02	2.01E-03	2.11E-02	3.92E-03	1.37E-02	2.49E-03
ETV vs.1μM	2.22E-02	2.33E-05	3.01E-04	1.13E-04	4.00E-03	2.11E-04
ETV vs.10μM	8.05E-01	4.77E-01	6.66E-03	4.06E-02	3.27E-02	3.49E-03

Abbreviation; Grey shade denoted significant difference between arbitrary two groups (p<0.05).

Supplementary table 2.

List of genes of their expression level was changed commonly with treatment AGI7 and AGI14 and the genes which recognized promoter region of E2F, E2F3, and Sp1

up regulation					
**gene symbol**	**E2F (M00803_0)**	**E2F (M00803_1)**	**gene symbol**	**E2F (M00803_0)**	**E2F (M00803_1)**
10-Sep	*	*	MIR1914	*	*
AADACP1	*	*	MIR6087		
ABCC4	*	*	MSC	*	*
ADAMTSL3	*	*	MSH2	*	*
ADPRM			MTHFS	*	*
AIRE	*	*	MYLK4	*	*
ALDH18A1	*	*	NAA16	*	*
ALDH1L2	*	*	NAB2	*	*
ALKBH1	*	*	NACC1	*	*
ANKS4B			NADSYN1	*	
ARMCX2			NALCN	*	*
ASTE1	*		NBPF3	*	*
ATIC	*	*	NDUFAF2	*	*
BAZ2A	*	*	NEURL1B	*	*
BMP8B	*	*	NICN1	*	*
BOLA1	*		NME6	*	*
BRI3BP	*	*	NOL6	*	*
C10orf10	*	*	NR0B1	*	*
C16orf74	*	*	NRTN	*	*
C17orf58	*	*	NSDHL	*	
C1orf167			NUBPL	*	*
CANX	*	*	NUDT18	*	*
CAPG	*	*	NVL	*	*
CAV1	*	*	OSTC	*	*
CCDC88B			PARP11		
CD36			PCYT2	*	*

CDR2	*	*	PGPEP1	*	*
CFH			PIGP	*	*
CHRDL2	*	*	PLS1	*	*
CKMT2-AS1	*	*	PMS1	*	*
CLIC6	*	*	PNRC2	*	*
COX10	*	*	PODXL	*	*
CSRNP3			POLI	*	*
DDX18	*	*	POLR3F	*	*
DHX29	*		PRDX4	*	*
DNAJC12	*	*	PRMT9	*	*
DNAJC22			PYCRL	*	*
DPY19L1	*	*	RAC3	*	*
DPY19L2P2	*	*	RBMXL1	*	*
DPYD	*	*	RFXANK	*	*
ELK3	*	*	RIPK3	*	
EMC3-AS1			RLTPR	*	*
ENTPD5	*	*	RNF13	*	*
EPS15	*	*	RNLS	*	*
ERCC4	*	*	RSBN1L	*	*
ERCC6	*	*	RTN4R	*	*
ESD	*		SERPINA10		
ETV6	*	*	SHISA4	*	*
FABP5	*	*	SHPRH	*	*
FAM185A	*	*	SHROOM3		
FAM46C	*	*	SIX5	*	*
FBXL14	*	*	SLA2	*	
FGF18	*	*	SLC22A9		
FGFR2	*	*	SLC35B2	*	*
FGG			SLC45A3	*	*
FHOD1	*	*	SLC6A3	*	*
FIGNL1	*	*	SMAD5	*	*
FXN	*	*	SMOX	*	*

GALK1	*	*	SNORA62		
GATA2	*	*	SPATA18	*	*
GGACT	*	*	SPG11	*	
GINS2	*	*	SSRP1	*	*
GKAP1	*	*	SULT2A1	*	
GLIS3-AS1			SYTL3	*	*
GPC2	*		TAF1B	*	*
GUCY1A3	*	*	TBC1D5	*	*
HCFC1	*	*	TBX19		
HEXA-AS1	*	*	TDP1	*	*
HNRNPH3	*	*	TDRD3	*	*
HS3ST2	*	*	TENM3	*	*
HSD17B1	*	*	THNSL1		
HSP90B1	*	*	THNSL2	*	*
IGF1R	*	*	TMEM154	*	
IL12A-AS1			TMEM260		
IL1RL2	*	*	TMEM50A	*	*
IL22RA1	*		TRIM8	*	*
INTS6-AS1	*	*	TSPAN7	*	*
IRF2BPL	*	*	TSR1	*	*
ITGAV	*	*	TXNL4B	*	*
KANTR	*	*	UBR5-AS1		
KBTBD8	*	*	UFM1	*	*
KCMF1	*	*	URGCP	*	
KLF13			USH2A		
KNDC1			USP10	*	*
LINC00294			USP12-AS2		
LINC01485			WDR36		
LINC01488			YPEL4	*	*
LIPT2	*		ZBED5-AS1	*	*
LOC100270746			ZHX1-C8orf76	*	*
LOC100499484	*	*	ZNF227	*	*

LOC100506022			ZNF260	*	*
LOC101928994	*	*	ZNF33B	*	
LTA4H	*		ZNF441	*	
LUZP1	*	*	ZNF562	*	
MED24			ZNF805	*	*
MFAP4	*		ZNF830	*	*
MID2	*	*	ZNF865	*	*
**down regulation**					
**gene symbol**	**Sp1 (M00196_0)**	**E2F3 (M02089_1)**	**gene symbol**	**Sp1 (M00196_0)**	**E2F3 (M02089_1)**
ACCS		*	LY6K	*	*
ACOT6			LZTFL1	*	*
ADAMTS17	*	*	MAFB	*	*
ADGRB2	*	*	MEIS3P1		
AMZ2P1	*	*	METTL8	*	*
ARID2	*	*	MFSD4	*	*
ARL4C	*	*	MGARP	*	*
ARMCX5			MIR29C		
ASIC1	*	*	MIR4665	*	*
ATP2A1	*	*	MUC2		
ATP7A	*	*	NAPRT	*	*
ATXN2L	*	*	NEBL	*	*
BBS7	*	*	NEDD1	*	*
BRAF	*	*	NEIL2	*	
BTN3A3			NID2	*	*
BUB1B			NPDC1	*	*
C11orf49	*	*	NPIPB11		
C12orf4	*		NRDE2	*	*
C12orf65	*		NSFL1C	*	*
C15orf52	*		NT5C2	*	*
C18orf42	*	*	NUP35		
C21orf33	*	*	OCA2	*	*

CA9	*	*	OCLM		
CAPS			OR7E2P		
CBY3			PACS1	*	*
CCDC59	*	*	PATL1	*	
CCHCR1	*	*	PCBD2	*	*
CCNG2	*	*	PCNXL2		*
CDCA7	*	*	PCSK1		*
CDH3	*	*	PEMT	*	*
CETN4P			PEX19	*	
CFAP69	*	*	PHF12	*	*
CHRM3	*	*	PIK3IP1		
CLIC2			PLAGL1	*	*
CNTNAP4			PLCH2	*	*
COL17A1			PLCL2	*	*
COL4A5	*		PLEKHB2	*	*
CTD-2297D10.2			PLEKHM2	*	*
DLL3	*	*	PLS3-AS1	*	*
DOK7	*	*	PPP1R12C	*	*
DTD1	*	*	PSMB8-AS1	*	
DVL1	*	*	PSMD7	*	*
EFHC1	*	*	PTCD1	*	*
ENTPD1			PTPN23	*	*
ENTPD1-AS1	*	*	RAET1E		*
EXOSC5			RERGL		
F13A1	*		RMDN3	*	*
FAM228B	*	*	RNF146	*	*
FAM3A	*	*	RNU4ATAC	*	*
FAM76A	*	*	RPL23AP64		
FLJ10038		*	RPS6KL1	*	*
FOXL2	*	*	RPUSD1	*	*
FPGT	*		RRAGB	*	*
FSCN1	*	*	RTP3	*	*

GABRE	*	*	SAMD11	*	*
GALM			SCAMP5	*	*
GATA6	*	*	SERPINA12		
GATSL2	*	*	SETD4	*	*
GEN1	*	*	SGSM3	*	*
GLUD1P3			SLC17A5	*	*
GPR108	*	*	SLC30A3	*	*
GPRC5A	*	*	SLC35G3	*	*
GRASP	*	*	SLC44A2	*	*
GRIP1	*	*	SLC5A12	*	
HDDC2	*		SLFN12		
HEBP1	*	*	SLX4	*	*
HERC5	*	*	SMARCA4	*	*
HPSE	*	*	SMC2	*	*
ID2	*	*	SNORA66		
IFT52	*	*	SNORD116-24		
IGSF9	*	*	SNORD52	*	*
IL11	*	*	SNORD76		
ING4	*		STARD4-AS1	*	
INMT	*		STARD8		*
ITGAE	*	*	STK17B	*	*
JAKMIP3			TAF1A-AS1		
KCNJ5	*	*	TBKBP1	*	*
KIAA1549	*	*	TCF7L1	*	*
KIFC3			TCIRG1	*	*
LAMA3	*		TIRAP	*	*
LDLRAD2			TMEM132A	*	*
LIN54	*	*	TMEM200A	*	*
LINC00176			TMEM223	*	
LINC00319	*		TPPP	*	*
LINC00470			TRIM36	*	*
LINC00593			TSC2	*	*

LINC00598	*		TSPAN15		
LINC00702			TTC3P1		
LINC00705			TTN		
LINC00899			UPK1B	*	*
LINC00908			UTS2B	*	
LINC01002			VCPKMT	*	*
LINC01125			VPS11	*	
LINC01564			ZBTB16	*	*
LOC100129148			ZBTB22	*	*
LOC101928068			ZBTB34	*	*
LOC101928140			ZFP36L1	*	*
LOC101929295			ZFP69	*	
LOC339874	*	*	ZFP90	*	*
LOC399815			ZMYND8	*	
LOC401127			ZNF137P		
LOC729737			ZNF407	*	*
LRRC8D	*	*	ZNF594	*	
LSMEM2	*	*	ZNF841	*	*

Abbreviation up: the expression level of genes in PXB cells treated with AGI7 and AGI14 is commonly upregulated as compared that in PXB cell, asterisk denoted the genes recognized promoter region of E2F or Sp1.

Supplementary table 3. List of sequence of siRNA

siRNA for Sp1
hSp1 #1
5'-GCXCAAACGUACACACACAtt
5'-UGUGUGUGUACGUUUGUGCtt
hSp2 #2
5'-CCUCUUUGAAGGUGGGAAAtt
5'-UUUCCCACCUUCAAAGAGGtt
hSp1 #3
5'-CAGAAGAGACUGAUCCAAAtt
5'-UUUGGAUCAGUCUCUUCUGtt
siRNA for E2F3
hE2F3 #1
5'-GGGCAAAGGAAGAGCUGCAtt
5'-UGCAGCUCUUCCUUUGCCCtt
hE2F3 #2
5'-GAACAAGGCAGCAGAAGUGtt
5'-CACUUCUGCUGCCUUGUUCtt
hE2F3 #3
5'-CCAAUGUGUUUGUGAGUUUtt
5'-AAACUCACAAACACAUUGGtt

A

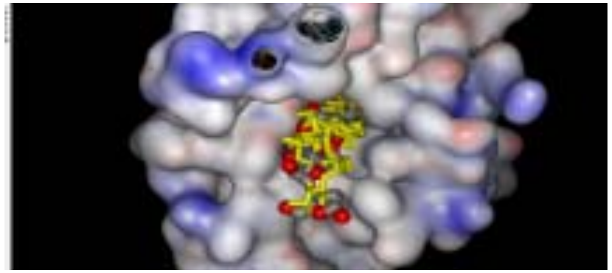

AGI1

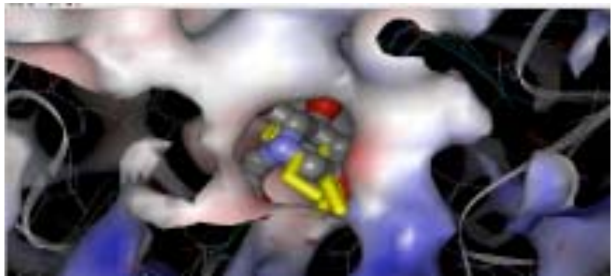

AGI2

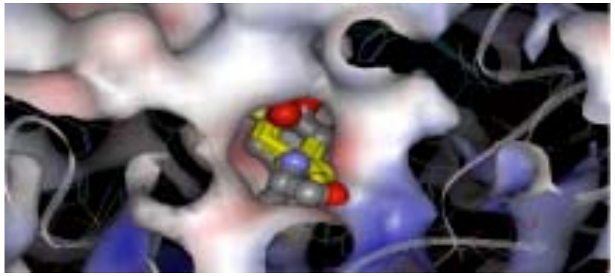

AGI3

B

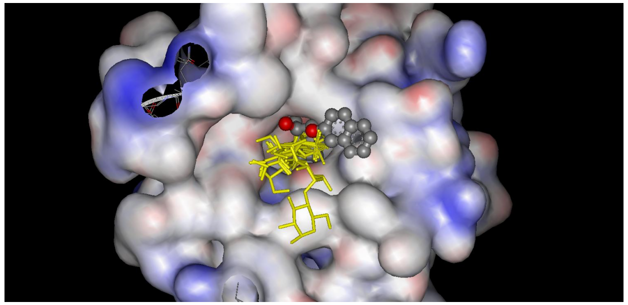

AGI5

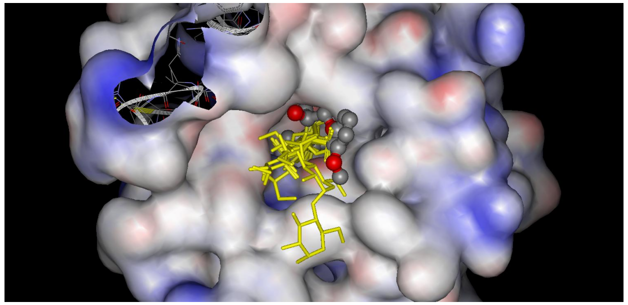

AGI6

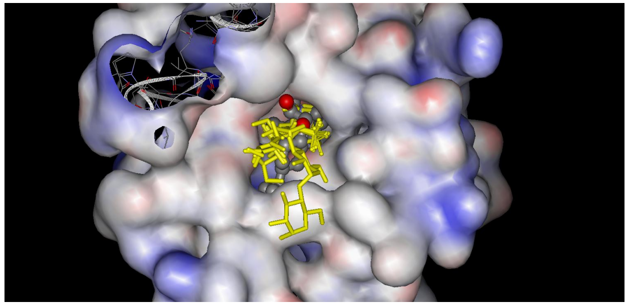

AGI7

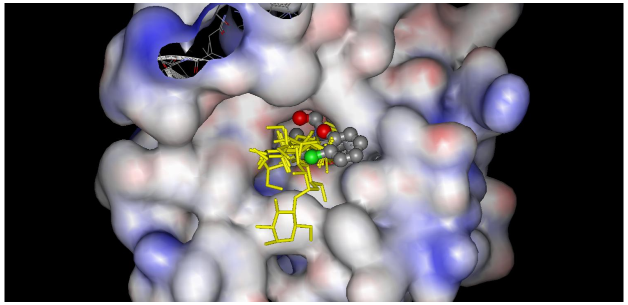

AGI13

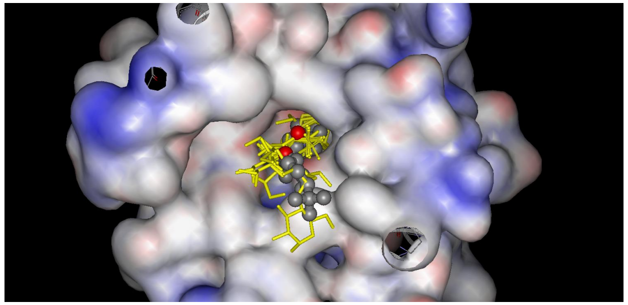

AGI14

A

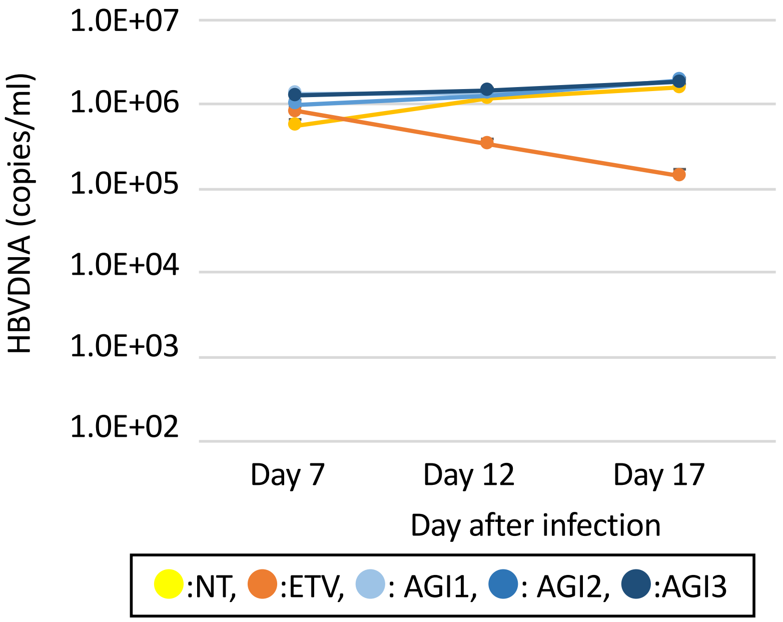

B

Cell toxicity	d7	d12	d17
**AGI1**	○	○	○
**AGI2**	○	○	○
**AGI3**	○	○	○

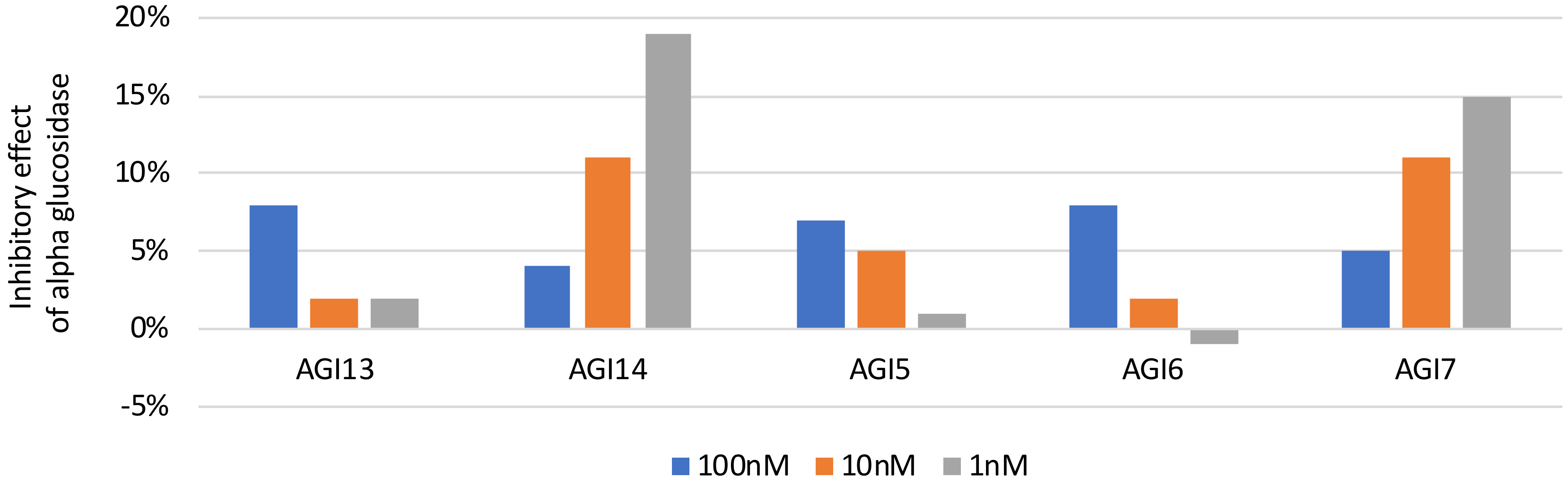
Inhibitory effect
of alpha glucosidase
20%
15%
10%
5%
0%
-5%
AGI13
AGI14
AGI5
AGI6
AGI7
100nM
10nM
1nM

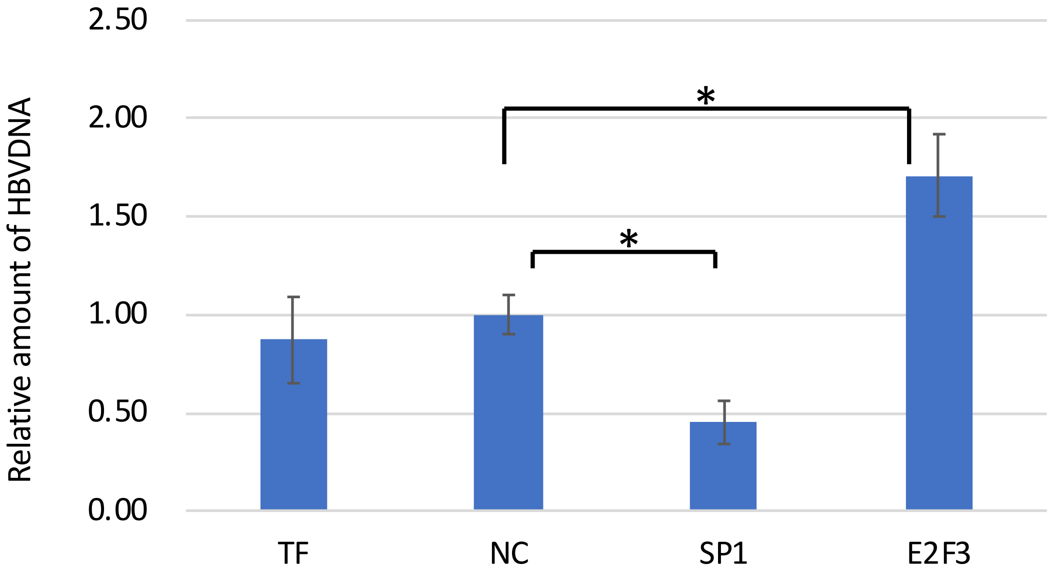
Relative amount of HBVDNA
2.50
2.00
1.50
1.00
0.50
0.00
TF
NC
SP1
E2F3
*
*

*: p<0.05
